# Supplementary material for: Subtype-specific secretion of extracellular vesicles by LRRK2 and Rab GTPases under lysosomal stress
Source: iScience. 2026 Aug 13;29(9):117203. doi: 10.1016/j.isci.2026.117203 (PMC13495093; doi:10.1016/j.isci.2026.117203)
Supplement: Supplementary file 1 [file mmc1.pdf]

iScience, Volume *29*

Supplemental information

Subtype-specific secretion of extracellular vesicles by LRRK2 and Rab GTPases under lysosomal stress

Maria Sakurai, Tomoki Kuwahara, Shoichi Suenaga, Sho Takatori, Taisuke Tomita, Tammy Shalit, Elizabeth Tengstrand, Frank Hsieh, and Takeshi Iwatsubo

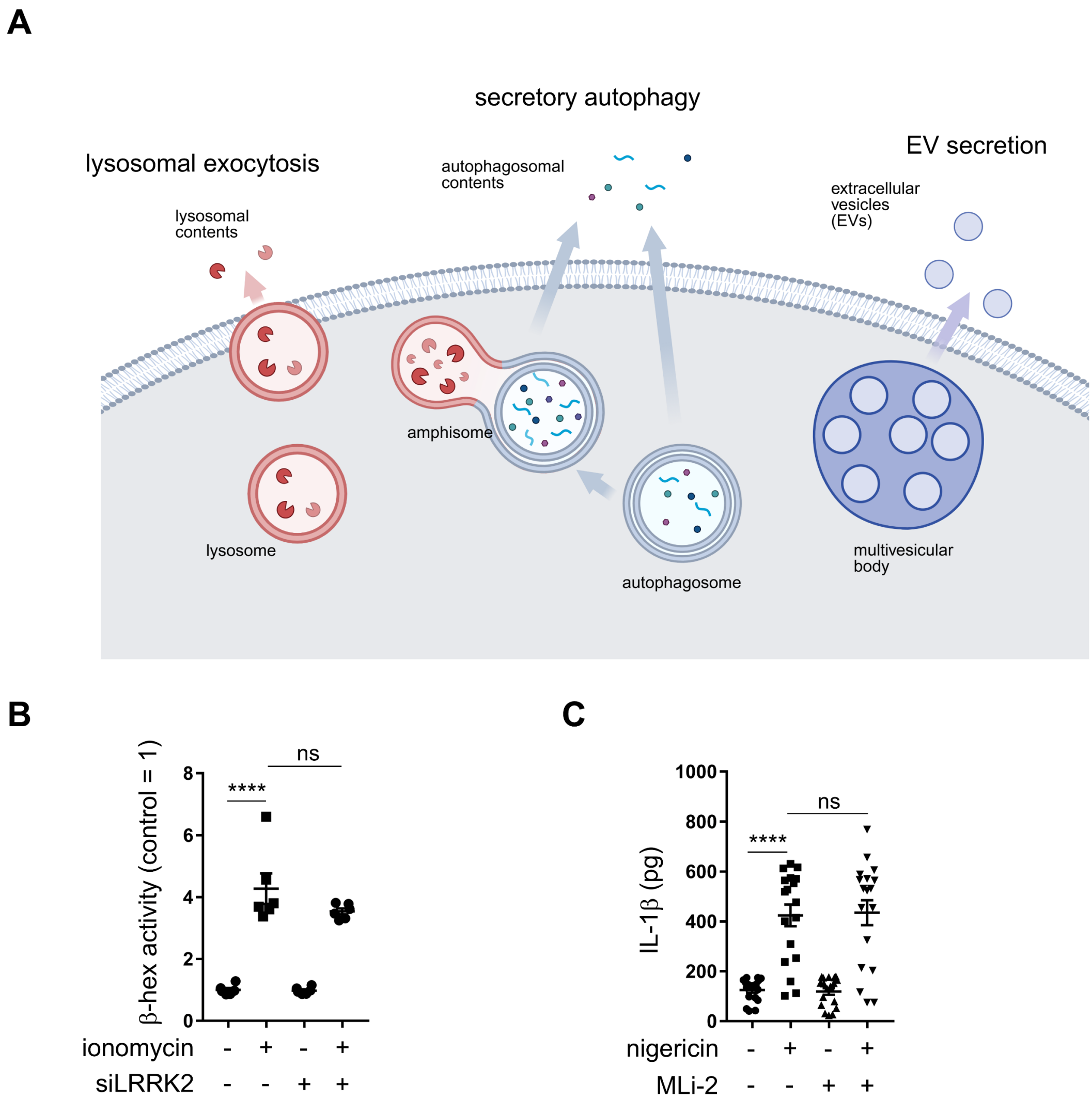

Figure S1. Analysis of LRRK2 involvement in the known endolysosomal secretory pathways, related to Figure 1.

(A) Three known endolysosomal secretory pathways that differ in membrane dynamics but are suggested to involve ATG8 conjugation system proteins acting upstream of LRRK2. **(B)** Assessment of lysosomal exocytosis by measuring β-hex activity in the medium of RAW264.7 cells pretreated with siLRRK2 and then treated with ionomycin. n = 6, mean ± SEM, one-way ANOVA with Tukey's test. **** $p < 0.0001$. **(C)** Assessment of secretory autophagy by measuring IL-1b levels in the medium of RAW264.7 cells treated with or without nigericin and MLi-2. n = 19, mean ± SEM, one-way ANOVA with Tukey's test. **** $p < 0.0001$.

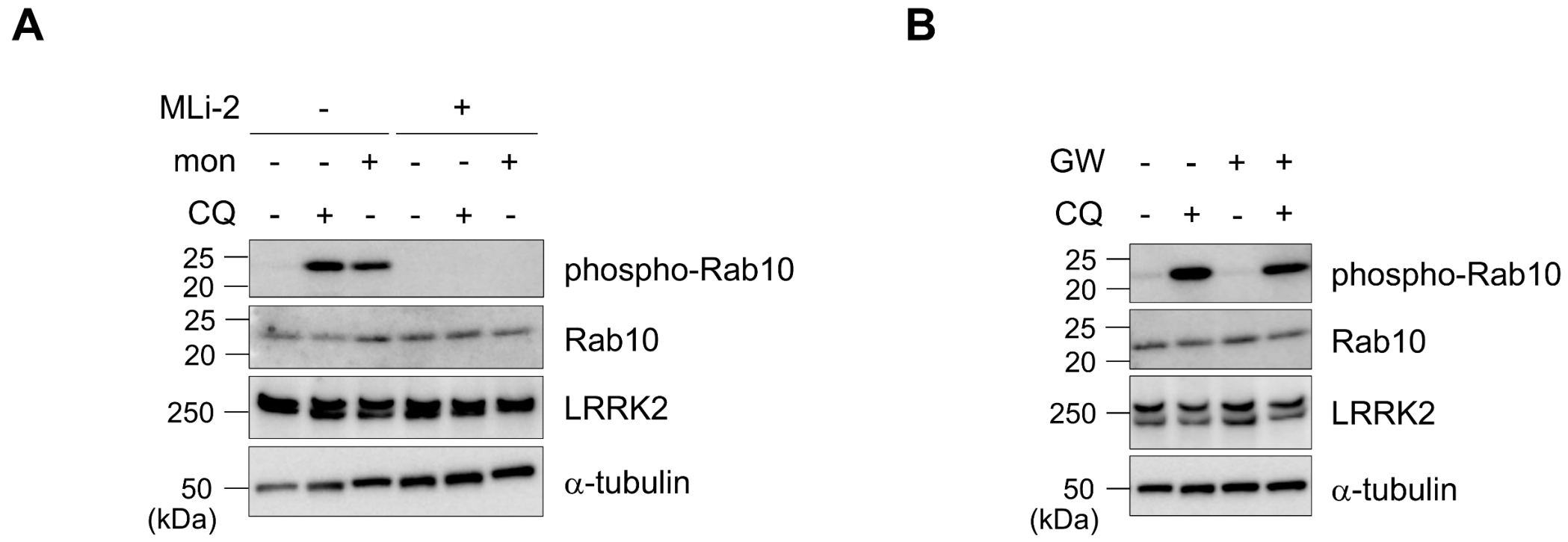

Figure S2. LRRK2 activation upon CQ or monensin treatment and its independence from GW4869 treatment, related to Figures 1 and 2.

(A) Immunoblot analysis of cell lysates derived from RAW264.7 cells treated with or without CQ, monensin and MLi-2. **(B)** Immunoblot analysis of cell lysates derived from RAW264.7 cells treated with or without CQ and GW4869.

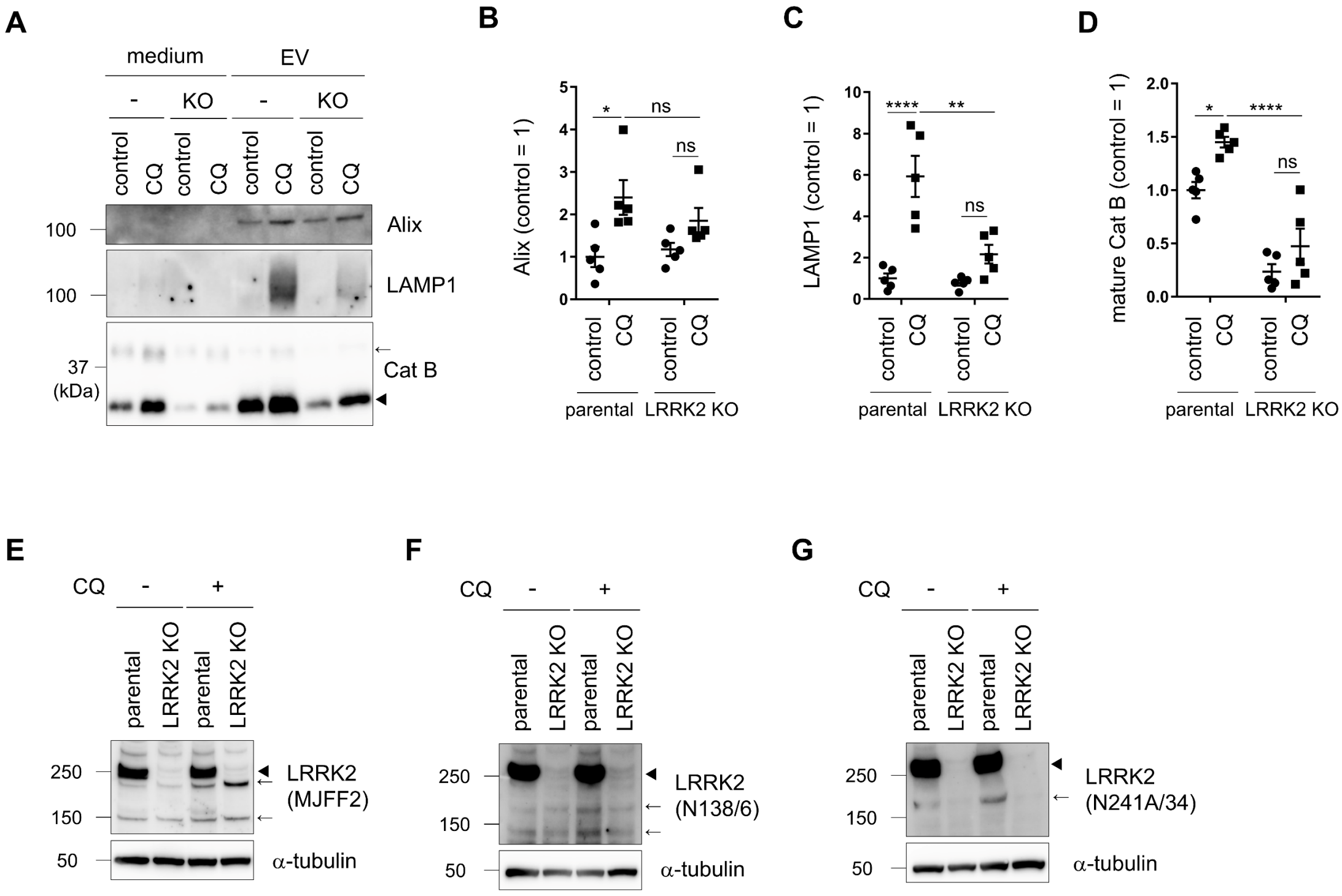

Figure S3. Analysis of CQ-induced secretion of EVs using LRRK2 KO and parental RAW264.7 cells, related to Figure 1.

(A) Immunoblot analysis of medium and EV fractions derived from LRRK2 KO and parental RAW264.7 cells treated with or without CQ. **(B-D)** Quantification of Alix (B), LAMP1 (C) and mature Cat B (D) in EV fractions, as shown in A. n = 5, mean ± SEM, two-way ANOVA with Tukey's test. *$p < 0.05$, **$p < 0.01$, ****$p < 0.0001$. **(E-G)** Immunoblot analysis of LRRK2 KO and parental RAW264.7 cells for the confirmation of knockout of LRRK2 protein expression using anti-LRRK2 antibodies MJFF2 (E), N138/6 (F) and N241A/34 (G). Arrowheads indicate full-length LRRK2; arrows indicate either LRRK2 fragments or non-specific bands.

A **B**

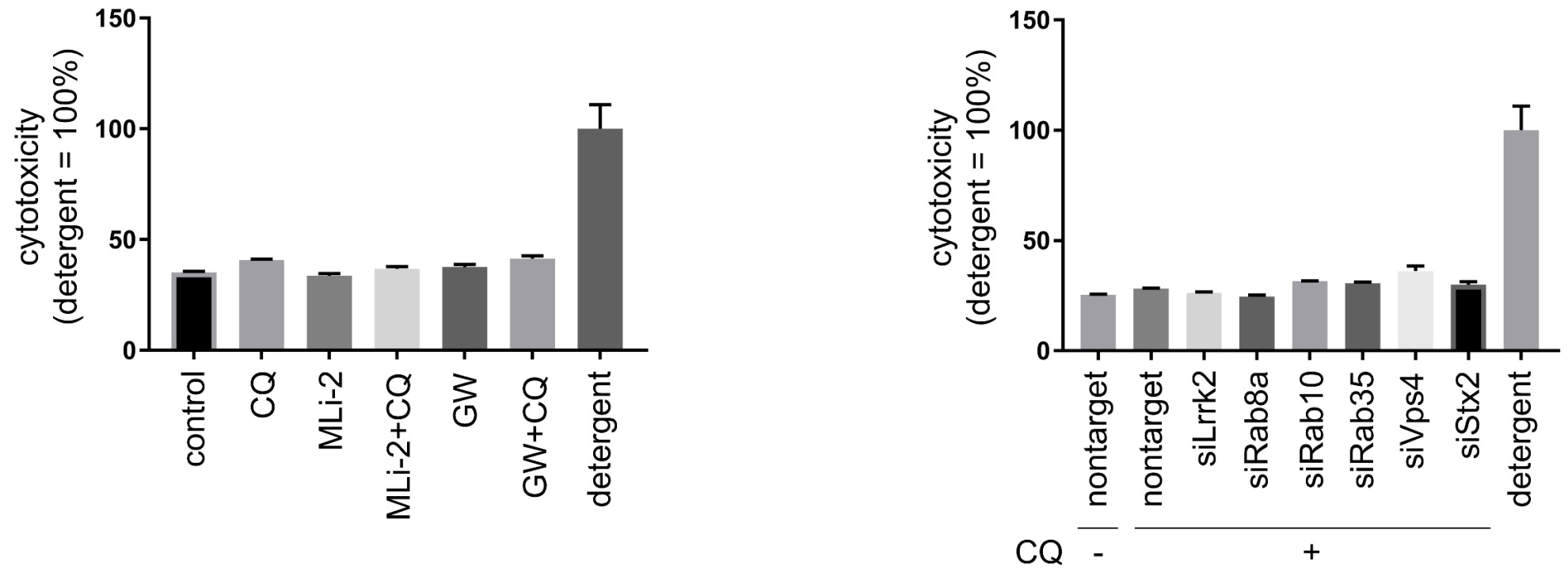

Figure S4. Lack of cytotoxicity following drug treatment or knockdown, as assessed by LDH activity measurement, related to Figures 1, 2 and 5-7.

(A) Cytotoxicity was assessed by measuring LDH activity in culture media of RAW264.7 cells treated with CQ, MLi-2 or GW4869. n = 3, mean ± SEM. **(B)** Cytotoxicity was assessed by measuring LDH activity in culture media of RAW264.7 cells treated with the indicated siRNAs as well as CQ. n = 3, mean ± SEM.

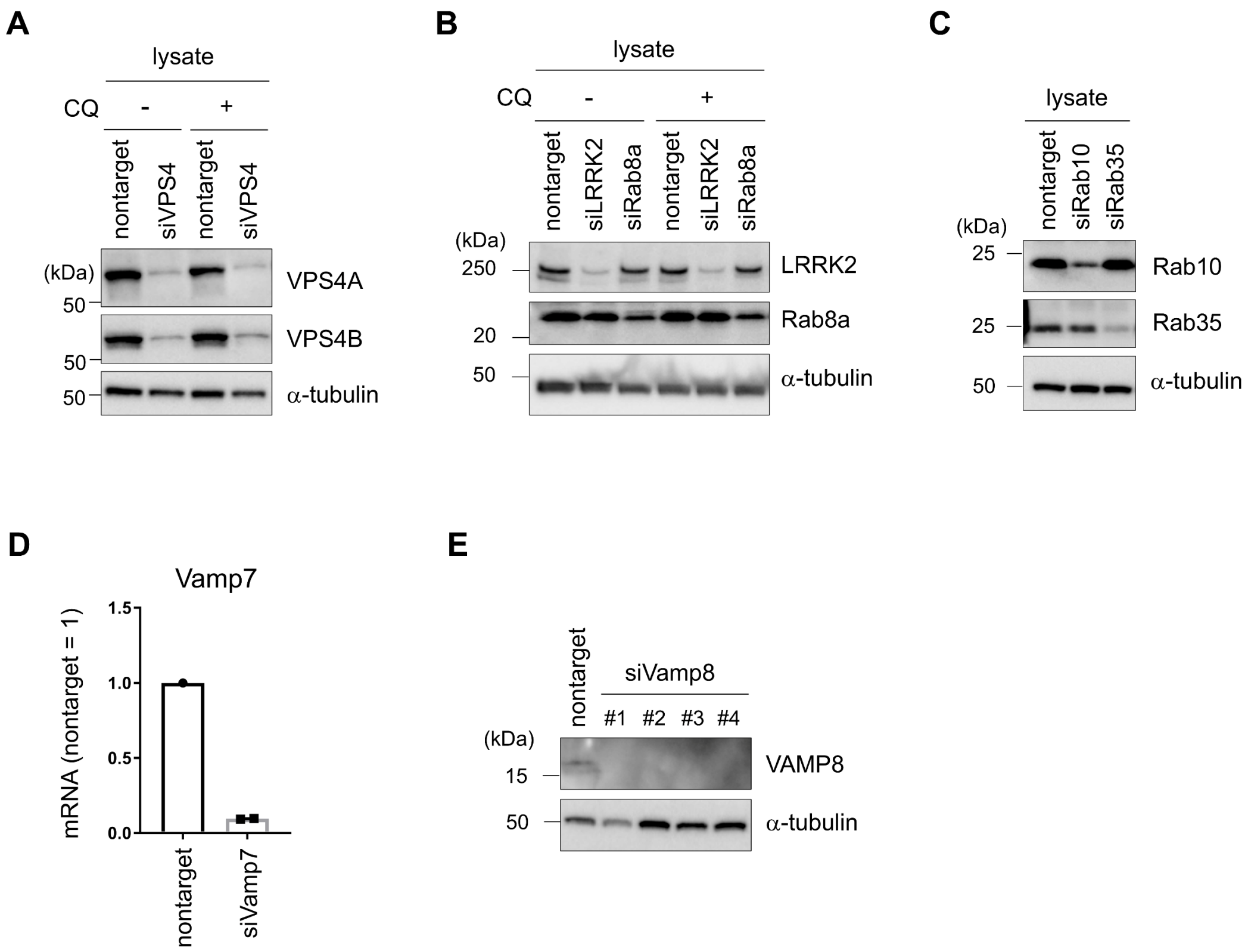

Figure S5. Confirmation of the knockdown efficiencies, related to Figures 5, 6 and 7.
(A-C) Immunoblot analysis of protein knockdown efficiencies of VPS4A/B (A), LRRK2 (B), Rab8a (B), Rab10 (C) and Rab35 (C) in RAW264.7 cells treated with each siRNA as indicated. **(D)** The mRNA expression levels of Vamp7 in RAW264.7 cells treated with siVamp7 or non-target siRNA, as quantified by qRT-PCR. Data represent mean of two independent experiments. **(E)** Immunoblot analysis of protein knockdown efficiencies of VAMP8 in RAW264.7 cells treated with four different Vamp8 siRNAs.

Table S1. List of genes targeted for knockdown screening, related to Figure 7.

No.	gene	protein
1	Acbd3	acyl-CoA binding domain containing 3
2	Alg2	ALG2 alpha-1,3/1,6-mannosyltransferase
3	Anxa7	annexin A7
4	Arf6	ARF GTPase 6
5	Arpc2	actin-related protein 2/3 complex subunit 2
6	Arpc4	actin-related protein 2/3 complex subunit 4
7	Ccdc93	CCC complex scaffolding subunit CCDC93
8	Cdc42	cell division cycle 42
9	Chmp1a	charged multivesicular body protein 1A
10	Chmp3	charged multivesicular body protein 3
11	Chmp6	charged multivesicular body protein 6
12	Clint1	clathrin interactor 1
13	Coro1b	coronin 1B
14	Coro1c	coronin 1C
15	Coro7	coronin 7
16	Dnajc5	DnaJ heat shock protein family (Hsp40) member C5
17	Eea1	early endosome antigen 1
18	Ehd1	EH domain containing 1
19	Ehd4	EH domain containing 4
20	Fam129a	niban apoptosis regulator 1
21	Fam129b	niban apoptosis regulator 2
22	Fam134b	reticulophagy regulator 1
23	Fam184b	family with sequence similarity 184 member B
24	Fam65c	RIPOR family member 3
25	Gopc	golgi associated PDZ and coiled-coil motif containing
26	Gphn	gephyrin
27	Hgs	hepatocyte growth factor-regulated tyrosine kinase substrate
28	Mapk8ip3	mitogen-activated protein kinase 8 interacting protein 3
29	Mvb12a	multivesicular body subunit 12A
30	Myo1d	myosin ID
31	Nsf	N-ethylmaleimide sensitive factor, vesicle fusing ATPase
32	Pacsin2	protein kinase C and casein kinase substrate in neurons 2
33	Pdcd6ip	programmed cell death 6 interacting protein
34	Rac1	Rac family small GTPase 1
35	Scamp5	secretory carrier membrane protein 5
36	Sec22a	SEC22 homolog A, vesicle trafficking protein
37	Snap23	synaptosome associated protein 23
38	Snf8	SNF8 subunit of ESCRT-II
39	Snx27	sorting nexin 27
40	Snx6	sorting nexin 6
41	Spag9	sperm associated antigen 9
42	Sqstm1	sequestosome 1
43	Stx2	syntaxin 2
44	Stx3	syntaxin 3
45	Stx4a	syntaxin 4
46	Stx6	syntaxin 6
47	Stxbp1	syntaxin binding protein 1
48	Syt5	synaptotagmin 5
49	Syt7	synaptotagmin 7

50	Tmem175	transmembrane protein 175
51	Tmem59	transmembrane protein 59
52	Tsg101	tumor susceptibility 101
53	Usp19	ubiquitin specific peptidase 19
54	Vamp2	vesicle associated membrane protein 2
55	Vamp3	vesicle associated membrane protein 3
56	Vamp7	vesicle associated membrane protein 7
57	Vps11	VPS11 core subunit of CORVET and HOPS complexes
58	Vps16	VPS16 core subunit of CORVET and HOPS complexes
59	Vps25	vacuolar protein sorting 25 homolog
60	Vps26b	VPS26 retromer complex component B
61	Vps28	VPS28 subunit of ESCRT-I
62	Vps29	VPS29 retromer complex component
63	Vps35	VPS35 retromer complex component
64	Vps36	vacuolar protein sorting 36 homolog
65	Vps37a	VPS37A subunit of ESCRT-I
66	Vps39	VPS39 subunit of HOPS complex
67	Vps41	VPS41 subunit of HOPS complex
68	Vps51	VPS51 subunit of GARP complex
69	Vti1b	vesicle transport through interaction with t-SNAREs 1B
70	Washc4	WASH complex subunit 4
71	Wdr91	WD repeat domain 91
**Positive controls**		
72	Lrrk2	leucine rich repeat kinase 2
73	Atg16l1	autophagy related 16 like 1
